# A Genetic Algorithm Based One Class Support Vector Machine Model for Arabic Skilled Forgery Signature Verification

**DOI:** 10.3390/jimaging9040079

**Published:** 2023-03-29

**Authors:** Ansam A. Abdulhussien, Mohammad F. Nasrudin, Saad M. Darwish, Zaid Abdi Alkareem Alyasseri

**Affiliations:** 1 Centre of Artificial Intelligence, Faculty of Information Sciences and Technology, University Kebangsaan Malaysia, Bangi 50300, Malaysia; mfn@ukm.edu.my; 2 Information Technology Center, Iraqi Commission for Computers and Informatics, Baghdad 10009, Iraq; 3 Institute of Graduate Studies and Research, University of Alexandria, Alexandria 21526, Egypt; saad.darwish@alexu.edu.eg; 4 Information Technology Research and Development Center (ITRDC), University of Kufa, Najaf 540011, Iraq; zaid.alyasseri@uokufa.edu.iq; 5 College of Engineering, University of Warith Al-Anbiyaa, Karbala 56001, Iraq

**Keywords:** offline signature verification system, preprocessing, feature fusion, forgery detection, Arabic signature, one-class support vector machine

## Abstract

Recently, signature verification systems have been widely adopted for verifying individuals based on their handwritten signatures, especially in forensic and commercial transactions. Generally, feature extraction and classification tremendously impact the accuracy of system authentication. Feature extraction is challenging for signature verification systems due to the diverse forms of signatures and sample circumstances. Current signature verification techniques demonstrate promising results in identifying genuine and forged signatures. However, the overall performance of skilled forgery detection remains rigid to deliver high contentment. Furthermore, most of the current signature verification techniques demand a large number of learning samples to increase verification accuracy. This is the primary disadvantage of using deep learning, as the figure of signature samples is mainly restricted to the functional application of the signature verification system. In addition, the system inputs are scanned signatures that comprise noisy pixels, a complicated background, blurriness, and contrast decay. The main challenge has been attaining a balance between noise and data loss, since some essential information is lost during preprocessing, probably influencing the subsequent stages of the system. This paper tackles the aforementioned issues by presenting four main steps: preprocessing, multifeature fusion, discriminant feature selection using a genetic algorithm based on one class support vector machine (OCSVM-GA), and a one-class learning strategy to address imbalanced signature data in the practical application of a signature verification system. The suggested method employs three databases of signatures: SID-Arabic handwritten signatures, CEDAR, and UTSIG. Experimental results depict that the proposed approach outperforms current systems in terms of false acceptance rate (FAR), false rejection rate (FRR), and equal error rate (EER).

## 1. Introduction

A signature is one of the most important human attributes. It is often used as proof of identity on legal documents like bank checks, credit cards, and wills. An effective automatic system can handle many fraud issues and other daily crimes. There are two different kinds of signature verification scenarios: online and offline. An online signature verification system uses tablets, PDAs, iPads, and smartphones to evaluate the signature image. The system has a dynamic nature, operating on features such as writing, orientation, pen tip positions, momentum, velocity, pressure, etc. [1,2].

An offline verification validates signatures by employing an optical detector to collect signatures on paper. This approach contains static data such as inclination, boundary, signature length and altitude, baseline, pressure, and size [3]. Offline verification is more intricate than online verification due to the absence of dynamic parameter information. Moreover, in an OSV system, signatures are obtained from various devices, so the resolution of training and testing samples is not the same, resulting in intraclass variation [4]. The raw signature may contain additional pixels known as noises or may not be in perfect working order, necessitating preprocessing.

Additionally, variations in the original signer’s signature are related to document orientation, signature degeneration, illness, illegible signatures, pen width, age, etc. As a result, preprocessing is a fundamental stage to improve the input data or isolate raw data samples into a standard format appropriate for the feature extraction stage. Nevertheless, it is crucial to balance noise removal and data loss, since a certain amount of relevant information may be lost during preprocessing, impairing the accuracy of subsequent system stages [5]. Therefore, proposing an algorithm that can effectively remove noise while maintaining relevant information is crucial.

Generally, the handwritten signature verification system determines whether the query signature is genuine or forged [2]. There are three forms of forgery: random forgery, simple (unskilled), and skilled forgery. In random forging, the forger uses a signature without knowing the original user’s name or signature. This signature shape is distinct from the genuine signature. The simple forger knows the user’s name, but is uninformed of the signature’s pattern. In this case, the forms of the original and forged signatures may not be similar.

**Figure 1:**
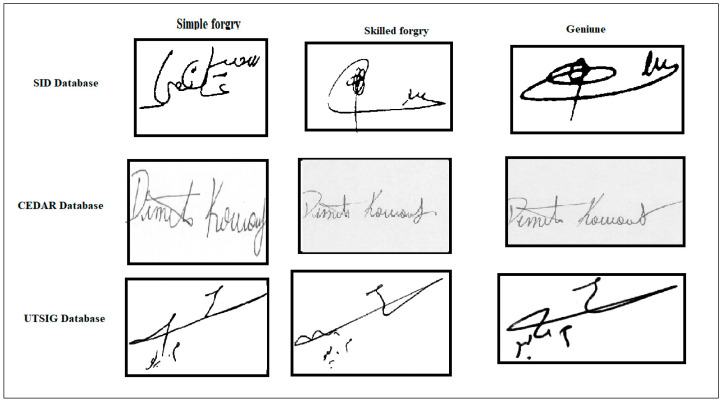
Sample of signatures.

On the other hand, the skilled forger learns the signature form and professionally mimics the signature with practice. This form of imitation is more challenging to detect since it is comparable to an authentic signature [6]. Figure 1 shows the samples of signatures. The most challenging aspect of the signature verification process is the substantial intra-class variance across signatures from the same individual, in contrast to low intra-class between forgery and genuine. Second, no comprehensive system can recognize all scripts.

According to the literature, signature verification systems have been the subject of substantial study in several languages, including English, Hindi, Bangla, and Chinese. However, studies on Arabic signatures are still limited, and improvement is slow because the language is more difficult to analyze than others. Arabic script is characterized by its cursive form, changing letter shapes, joining and non-joining characters, delayed strokes, and ligatures [7,8].

Recently, researchers have focused on two major processes: feature extraction and verification methods. Feature extraction methods depended on handcraft, such as statistical, geometric, structural, and automatic deep learning. In several cases, researchers have combined multiple methods to improve performance. Fusion is the process of integrating multiple patterns of components into a single matrix using a fusion approach, such as score-level fusion and high-priority index feature fusion [9].

The problem with earlier approaches was that they typically employed a combination of features without considering correlations and discriminants, such that the generated feature vector could not recognize a skilled forgery. In addition, fusion techniques were used to increase system complexity and computational time. Moreover, the current deep learning approaches are defective in the signature verification domain because deep training models necessitate large data samples and effort in selecting images suitable for learning construction [8]. Deep learning also necessitates substantial computer resources, such as expensive GPUs.

This research aims to propose an offline Arabic signature verification system that can recognize skilled forgery and genuine signatures at a highly accurate rate and low FAR, FRR, and EER. First, efficient preprocessing techniques such as image cropping, denoising, binarization, and removing stray isolated pixels are recommended to decrease noise while maintaining essential data.

The contributions of this study include the following:

1. Efficient preprocessing techniques are recommended to decrease noise while maintaining essential data.
2. Hybrid feature types have been proposed to solve the low inter-class variability between authentic and skilled forgery and the high intra-class variability in each individual’s signature.
3. The early serial concatenation fusion approach (ESCF) integrates multiscale information without prejudice complication.
4. Propose GA_OCSVM to improve feature selection and tackle the potential correlation between fused features
5. Settle the problem of unbalanced and restricted forgery samples by using one-class classification.

The extensive computing time and storage capacity are unnecessary for the proposed approach.

This paper has the following organization: Section 2 presents the related studies. The framework for the proposed signature verification system is given in Section 3. The experimental results and comparisons of the proposed methodology to previous research are presented in Section 4. The summary of the proposed work is presented in Section 5. The conclusion and recommendations for further work are provided in Section 6.

## 2. Related Works

Signatures are typically simple or unconventional, and have no distinct characteristics that are hard to lose or forget compared to other biometric features [10]. Consequently, signatures on checks, card payments, and legal documents are often used and accepted as evidence of authorship or approval. Signatures are currently authenticated in various environments [11]; however, the rapid progress of computer technology has attracted the attention of researchers in automated signature verification and authenticity detection. OSV has significantly evolved in the last decade; researchers have employed various methodologies and techniques to accomplish high performance, superior accuracy, and efficiency in offline signature verification. Signature verification methods are typically divided into template matching, statistical, and structural approaches [12].

In the template matching approach, the pattern of test signatures is compared with templates already stored in the database. Dynamic time warping (DTW) is the most often utilized for this purpose, ref. [13] proposed a crowdsourcing experiment to develop a human baseline for signature recognition and a new attribute-based automated signature verification system based on FDE analysis. The technique combines the DTW algorithm with an attribute-based approach to improve accuracy with 5% EER. The authors of [14] proposed a graph-based system for signature verification. This approach combines DTW with linear time graph dissimilarity to measure the polar graph embedding distance (PGEd) called structural DTW (SDTW). They used a sliding window approach to compare PGEd at various local positions on several subgraphs. The resulting distance matrix was used to find an optimal alignment between the sequences of subgraphs using DTW. The authors applied the proposed method to standard GPDS-75 and MCYT-75 datasets.

However, statistical models are employed in the vast preponderance of signature verification systems, such as distance-based classification, support vector machine (SVM), deep learning, and other classification techniques. The distance-based approach is one of the most straightforward and reliable approaches for identifying query and reference signatures because this approach lacks parameters and model training [15]. Nevertheless, distance-based techniques are not interested in the influence of general variability on distance and often exhibit random fluctuations of varying sizes. The most prominent methods used in this domain are Euclidean distance, city block distance, Chi-square distance, Manhattan distance, and Hamming distance. On the SUSIG dataset, a Hadamard transform-based technique was developed [16]. The Hadamard matrix was generated from the extracted features, and then the Euclidean and Manhattan distances were employed for feature comparison and verification.

In [17], the Euclidian distance was employed to compare stored and new feature vectors. The investigation used one hundred and eight signature samples from participants. Global thresholding was used to convert images to grayscale, and the median filter was utilized to remove noise. The Canny edge detector was employed to identify signature edges. Seven hundred moments of invariants were calculated for five samples, and the standard deviation was used to generate a feature vector.

Similarly, SVM and deep learning techniques such as convolutional neural networks (CNNs) are the most often used classifiers in OSV. SVM performs well in high-dimensional spaces, regardless of whether the dimensionality exceeds the sample quantity. It is memory efficient because it uses a subset of training images (support vectors) in the decision function [18]. However, SVM is mathematically and computationally complex. Ref. [19] used the SVM and shape correspondence approaches for signature verification. Pixels were correlated using an adaptive weight that included Euclidean and shape context distances. Plate spline transformation was used to convert the query signature plane to the reference signature plane. On the GPDS signature dataset, the system achieved an accuracy of 89.58%. The authors in [19] used a decision tree classifier and a Local Binary Pattern feature extraction. Two collected datasets with 100 and 260 authors were employed to evaluate the performance of the system. The system produced a FAR of 7.0% and 11% for simple and skilled forging signatures, respectively.

Moreover, the authors in [20] introduced a dynamic signature verification technique (DSVT) using mutual compliance (MC) between the security system and the biometric device. The security system was responsible for online and offline signature approval using personal inputs from the user. The signing bit, key, and size were used as security metrics to verify both modes using classifier learning. The verification was based on stored online/offline signatures using certificates provided for authentication.

The E-signature was conducted based on the user’s specific inputs. The user authenticity was examined based on stored online/offline signatures using certificates and authentication during manual sessions. A traditional tree classifier was used to distinguish the dynamic verification between online and offline signatures. The success rate of the suggested strategy was 0.893%, while the failure rate was 8.58%.

The [21] compared SVM with five machine-learning classifiers, i.e., boosted tree, random forest classifier (RFC), K-nearest neighbor, multilayer Perceptron, and naïve Bayes classifier, utilizing four image-based characteristics. The BHsig260 dataset (Bangla and Hindi) was used in the proposed work, which included signatures from 55 Hindi and Bangla users. The offline Hindi signature verification accuracy using MLP with 20 sample sizes was 72.3%. The accuracy for Bangla was 79% using RFC with two signature samples, while KNN and SVM obtained above 92%.

In addition, various deep-learning techniques have been proposed for online and offline signature verification. In the offline signatures system, ref. [22] employed CNNs in a two-stage method. Feature representations were learned in the writer-independent phase by discriminatively training a CNN to identify authors. These CNN characteristics were then utilized for training writer-dependent classifiers (SVMs) to recognize differences between genuine and skilled signatures. Moreover, they tested this method using four distinct feature representation versions of AlexNet and VGG networks [23]. Kohonen neural networks were proposed to construct an offline signature verification system, which was a form of self-organizing map [24]. The intra-variability of an individual’s signatures is quantified using their competitive learning power. The proposed system achieved FAR and FRR for the genuine samples of 2.8% and 5%, respectively, for simple and random forgeries.

The researchers in [25] also used CNN to verify a Bengali handwritten signature. Two handwritten signature databases were used as experimental data for the training system. The first database contained 800 handwritten signature images of 40 students at the Fergana branch of the Muhammad al-Khwarizmi Tashkent University; each student had 10 genuine and 10 forged signatures. The second database was a public Bengali handwritten signature database, which included 100 people with 24 authentic and 30 skilled signatures. The average accuracy achieved for the first database was 90.04% on images of size 250 × 150, and 97.50% for the second database on images of size 250 × 150.

The researchers in [20] proposed an offline signature verification system using a multi-size assembled attention swin-transformer (MSAAST) network. The main modules included the resize, swin-transformer, and attention block. The signature images were resized to different sizes, including (224, 224), (112, 112), and (56, 56). Then, they were simultaneously put into the Patch-Embedded module and swin-transformer to extract and combine features. The cross-dataset strategies were used to improve the dataset; considering the generalization ability, CEDAR was utilized as a training dataset and evaluated in Bengali. Three databases were used to assess the model: CEDAR, Bengali, and Hindi. The training and testing datasets extended double, and images were concatenated in combination forms: genuine-genuine signature pairs (GGSP) or genuine-forgery signature pairs. The regularized dropout (R-Drop) strategy and adversarial methods were employed in the training phase to improve the verification performance. The authors used the R-Drop strategy to limit the model’s outputs and keep them in identical distributions even when the inputs were run through the model more than once. The accuracy metric significantly increased from 0.955 to 0.973. However, in the experiment on R-Drop, the dropout produced different outputs for the same input images each time.

Despite the tremendous achievements of deep learning in signature identification, one of the significant downsides of deep learning models is that they need a massive amount of labeled data for training to obtain a high level of accuracy. Most signature databases are limited (particularly concerning the number of original signatures per writer). This limitation faced the authors in [26], who used samples from the SVC 2004 and SigComp 2009 datasets to learn a convolutional neural network (CNN) followed by a recurrent neural network (RNN). The proposed model achieved low validation results due to the few samples used; the experiments showed 90.65% accuracy and 15.43% FAR.

In contrast to statistical representation models, structural (i.e., string, tree, and graph-based) techniques express the fundamental topological features of a handwritten signature in a highly natural and exhaustive form. This model compares the symbolic representation (trees, graphs, and strings) to database-stored models. However, this advantage comes at the expense of increased complexity in basic dissimilarity assessments [27]. The authors in [28] focused on dissimilarity-based graph embedding techniques for signature verification. It generated n-dimensional feature representations for graphs, which were then used to classify signatures. In an experimental assessment of the MCYT-75 and GPDS-960 benchmark datasets, the suggested technique achieved 10.67% EER and 16.53 EER using 10 references.

In addition to being accurate and secure, the signature verification process should be fast. Furthermore, signature verification is complicated since the distinctions used to discriminate are frequently precise. As a result, offline signature recognition is still open research. Table 1 displays an overview of related works and their respective outcomes.

## 3. Materials and Methods

**Figure 2:**
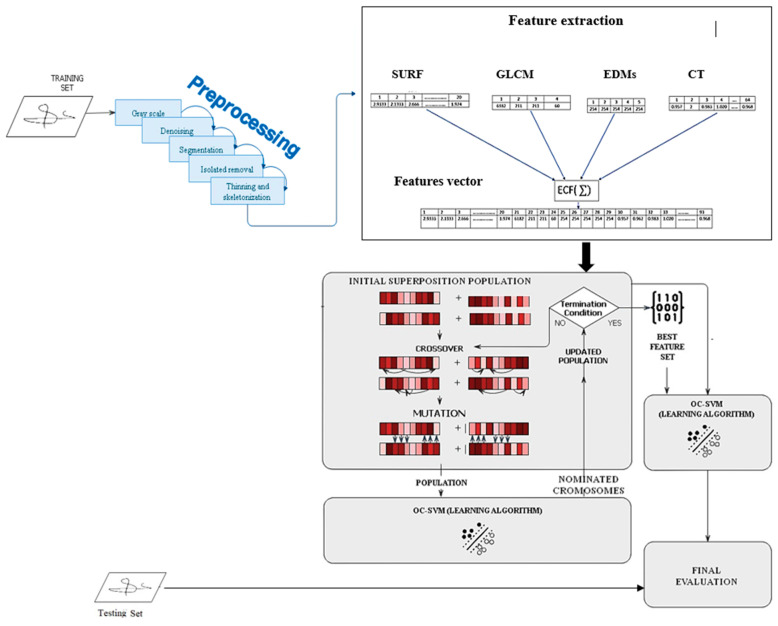
Flowchart of the proposed model.

The proposed model comprises five phases: preprocessing approaches, feature extraction, feature fusion, feature selection, and classification, as shown in Figure 2.

### 3.1. Preprocessing Phase

A review of the problems related to offline signatures may include noisy pixels or equipment that may not be in perfect working order. As a result, several preprocessing methods are presented to provide an improved image that can be utilized for subsequent phases without losing data.

#### 3.1.1. Image Conversion

The first step in the proposed approach is to convert an RGB image to a grayscale image, which is required to decrease system complexity and processing time because grayscale images are simpler to modify than RGB images.

#### 3.1.2. Noise Reduction

The scanner or the paper backdrop might produce noise in a scanned image; the image may become fuzzy due to insufficient illumination and stained regions, such as dots and speckles. The image filtering technique improves the image by converting irrelevant brightness information into valuable data, and is easily understandable and concentrated on machine interactions. The median filtering (MF) strategy is utilized in this work to remove noise from the signature image. The MF technique [40] is a statistically based nonlinear method for reducing image noise. Applying a linear low-pass filter is the preferred approach for smoothing, which is the appropriate method in a static signature. The MF has the following two key advantages:

MF retains sharp edges, whereas low-pass linear filtering softens the edges.

MF is quite effective in smoothing down a noise spike.

MF retains the pertinent information of the image and changes the original grey value of each pixel to the median gray value of the area of the neighborhood. This filter reduces visual noise without causing edge blurring. The median is calculated by sorting the pixel values of the neighborhood window and substituting the considered pixel with the middle (*median*) value. The formula for the MF image D(x,y) of image I(m,n) is represented as (1).

(1) $$D\left(x,y\right)={median}_{(m,n)\in R_{xy}}\left\{I\left(m,n\right)\right\}$$

where m,n∈ center around the processed pixel (x,y).

#### 3.1.3. Binarization

This process transforms a grayscale image into a binary image. Image binarization is the earliest step of image processing and analysis. Pixels in an image are separated into two different areas, black and white. The main goal of image binarization is to be able to describe the difference between text in the foreground and text in the background. The thresholding technique is the simplest type of binarization. In thresholding, pixels are identified as foreground or background by comparing them to the maximum threshold value. However, determining the optimal threshold value for such signature text is challenging. Inaccurate estimation of the threshold value leads to the erroneous classification of pixels as foreground or background, which affects binarization results and the accuracy of signature authentication.

In this research, the backdrop of an image is estimated using the grayscale morphological method [41]. The contrast of the image text regions is boosted using the approximate background data. A recognition threshold value for image sections is determined by analyzing the histogram of the contrast image. In image processing, morphology can be applied to two types of pixel sets: objects and structural elements (SEs). Objects are described as collections of foreground pixels. SEs are created using both foreground and background pixels.

The size of the SEs is first determined and calculated using the histogram of the distance between consecutive edges. The morphological processing techniques include dilation and erosion. SEs generate both dilation and erosion by interacting with a collection of exciting pixels in an image. The SEs have a morphology and an origin. A⊕B denotes dilation, which is the collection of all shifts satisfying the condition in Equation (2):

(2) $$A⨁B=\left\{z|{\left(\hat{B}\right)}_{z}\cap A\neq \emptyset \right\}$$

where *A* is a set of foreground pixels, *B* is SEs, $\left(\hat{B}\right)$ is the reflection of the structuring B about its origin, followed by a shift by *z*, and z’s are foreground values (one’s). The erosion represented by the symbol A⊖B is defined as Equation (3):

(3) $$A⊖B=\left\{z|{\left(B\right)}_{z}\subseteq A\right\}$$

#### 3.1.4. Image Segmentation

Segmentation is used to extract the signature region from an image. This procedure decreases the processing time by deleting the excess pixels of the image. In this work, an automated segmentation technique is computed using the histogram; the region is automatically segmented based on pixel values. The histogram depicts the total amount of black-and-white pixels [42]. The distribution is horizontally and vertically separated. The white pixel’s highest point is utilized as a trimming reference. The image is divided in half to simplify the recovery of the starting and ending points.

Consequently, two points denote the beginning and end of cutting originating from the highest point. The horizontal histogram determines the starting and ending positions of the horizontal trim. Additionally, vertical cutting uses the vertical histogram to calculate the beginning and ending locations. Equations (4)–(6) generate histograms:

(4) $$H_{X}=\sum_{y=1}^{n}bim\left(x,y\right)$$

(5) $$H_{Y}=\sum_{x=1}^{m}bim\left(x,y\right)$$

(6) $$imcrop=\left\{\begin{array}{cc} {origin}_{image} & Xmax\;<\;X\;<\;X\min\;\;and\;Ymax\;<\;Y\;<\;Ymin\\ 0 & otherwise \end{array}\right.$$

bim denotes the binary image, while m and n indicate the matrix bim’s rows and columns, respectively.

#### 3.1.5. Stray Isolated Pixel Elimination

In some signatures, extra points caused by ink flowing unrelated to the signature may affect the original signature area. Consequently, the MATLAB function eliminates any connected components (objects) with less than 50 pixels from the binary image B(x,y). This procedure is known as an area opening, as shown in Equation (7).

(7) $$B_{new}\left(x,y\right)=\mathrm{bwareaopen}\;(B\left(x,y\right)$$

#### 3.1.6. Skeletonization and Thinning

**Figure 3:**
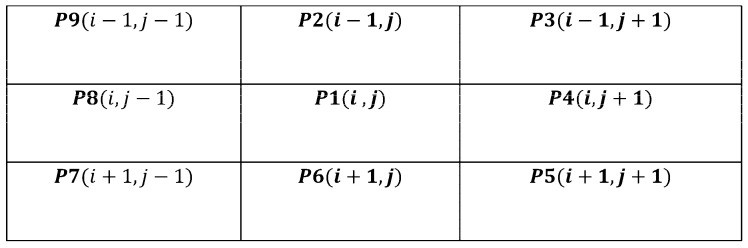
Designations of the pixels in a 3 × 3 window.

Thinning is an iterative process that results in skeleton production. This procedure minimizes the number of character characteristics to aid feature extraction and classification by erasing the width fluctuations of the pen. Applying a specific morphological operation to the binary image B, a fast parallel thinning method (FPT) removes inside pixels to leave an outline of the signature [43]. The FPT approach extracts the skeleton from an image by removing all contour points except those relevant to the skeleton. As illustrated in Figure 3, each point p(i,j) has eight neighbors.

**Figure 4:**
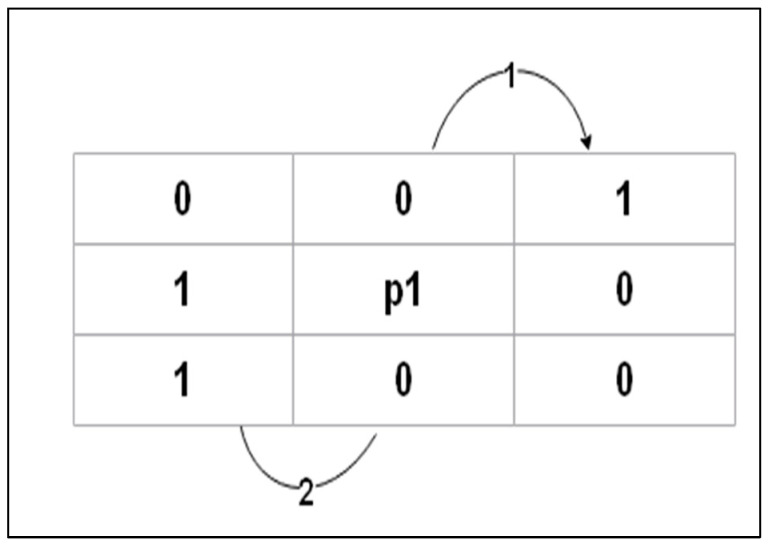
Counting the 01 patterns in the ordered set *P_2_*,…, *P_9_*.

Each iteration is separated into two subiterations to preserve the structure of the skeleton. See Algorithm 1 and Figure 4.
**Algorithm 1:** FPT
1: *A* (*P_1_*) is the number of (01) patterns in the ordered set *P_2_*, *P_3_*, *P_4_*, …., *P_8_*, *P_9_* that are the eight neighbors of *P_1_* 2: *B* (*P_1_*) is the number of nonzero neighbors of *P_1_* 3:B(P1) = $\sum_{i=2}^{9}P_{i}$ 4: Iteration 1: *P_1_* = 0                 If   2 ≤
*B* (*P_1_*) ≤ 6                If *A* (*P_1_*) = 1                    If  $P_{2}\times P_{4}\times P_{6}=0$                   If  $P_{4}\times P_{6}\times P_{8}=0$                             Else                                   *P_1_* = 1                                     *A* (*P_1_*) = 2 5: Iteration 2:                           *P_2_ × P_6_ × P_8_* = 0, *P_2_ × P_4_ × P_8_* = 0                   Keep the rest points 6: End

**Figure 5:**
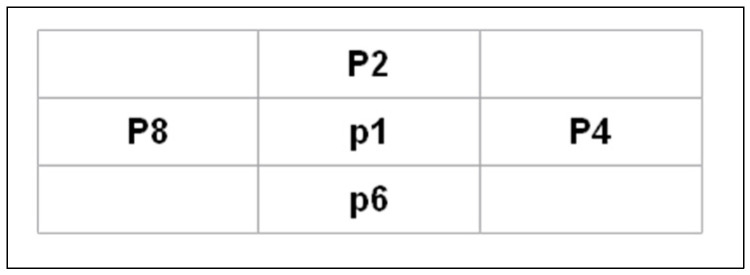
Locations of points that satisfy the conditions.

**Figure 6:**
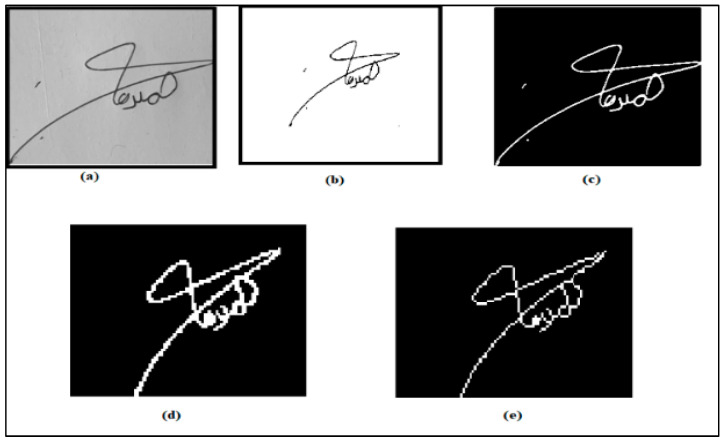
Preprocessing steps (**a**) grayscale image, (**b**) image denoising, (**c**) segmentation, (**d**) isolated removal, and (**e**) thinning and skeletonization.

Skeletonization is achieved by removing specific foreground pixels from a binary image through image thinning. Consequently, a collection of tiny arcs and curves portrays the signature pattern. The complement of the image is adjusted to make the signature bright and the background dark to skeletonize the original image. See Figure 5 and Figure 6.

### 3.2. Hybrid Feature Extraction

Feature extraction is a crucial step in the verification of a signature. The proposed Hybrid Statistical Feature Extraction (HSFE) technique extracts highly informative features by combining multiple types of features using three statistical approaches: interest point features, global and local texture features, and curvelet transformation features.

#### 3.2.1. Texture Feature

In image processing, the texture is described as a function of the spatial variation of the brightness intensity of the pixel. Image processing is the primary term to define objects or concepts in a given image. Texture analysis is critical in computer vision applications such as object recognition, surface defect detection, pattern recognition, and medical image analysis. This paper combines two statistical methods of edge direction matrices (EDMs) [44] and local binary pattern (*LBP*) [45] to extract texture attributes.

*LBP* features are also known as the texture operator for a grayscale image, which helps to characterize the spatial structure of the input image texture. Once the central pixel value is obtained, the pattern code can be computed by comparing these values to its neighborhoods. It can be expressed as Equation (1).

(8) $${LBP}_{N,R}=\sum_{n=0}^{N-1}{s(I_{n}-I_{g})}^{2N}$$

$$s(x)=\left\{\begin{array}{c} 1,x\geq 0\\ 0,x<0 \end{array}\right.$$

where $I_{g}$ denotes the gray value of the center pixel, $I_{n}$ represents the gray values of the circularly symmetrical neighborhood, and *N* denotes the total number of spaced pixels on a circle of radius *R*. The final texture feature employed in texture analysis is the histogram of the operator outputs (i.e., pattern labels) accumulated over a texture sample. The operator for grayscale and rotation-invariance texture description is shown in Equations (9) and (10).

(9) $${LBP}_{N,R}=\left\{\begin{array}{ccc} \sum_{n=0}^{N-1}s\left(I_{n}-I_{g}\right) & if & {ULPB}_{N,R}\leq 2\\ N+1 & & otherwise \end{array}\right.$$

where

(10) $${ULPB}_{N,R}=|s\left(I_{N-1}-I_{g}\right)-s\left(I_{0}-I_{g}\right)|+\sum_{n=1}^{N-1}\left|s\left(I_{n}-I_{g}\right)-s\left(I_{n-1}-I_{g}\right)\right|$$

**Figure 7:**
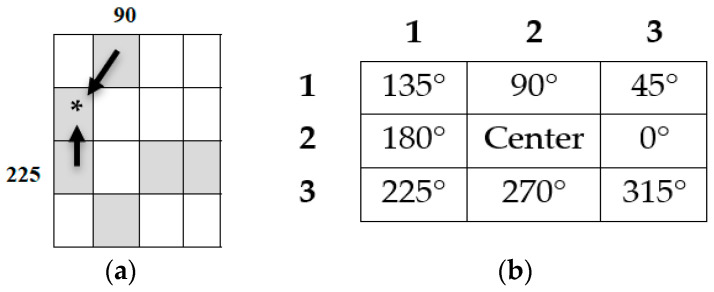
(**a**) Two neighboring edge pixels, (**b**) EDMs principal.

However, *LBP* cannot provide information about shape; that is, the spatial relationships of pixels in an image. As a result, *LBP* is combined with EDMS. The global features are the features that result from the shape of a signature contour [45]. EDMS is a feature extraction approach that detects the texture of a binary image I(x,y) based on edge-to-neighbor pixel relationships. Eight adjoining kernel matrices were applied, and each pixel was linked to two neighboring pixels. A connection was established between the edge pixel E(x,y) and its neighboring pixels, as illustrated in Figure 7a. The eight pixels were used to change the surrounding values into the position values, as shown in Figure 7b.

This approach is presented from two perspectives: first-order relationship (FOR) identification and second-order relationship (SOR) identification. Each cell in the FOR matrix has a location between 0 and 315 degrees, depending on the pixel neighborhood association. The relationship between the pixel values can be determined by computing the occurrence of the FOR values while considering the edge image of each pixel concerning two other pixels.

The relationships are sorted according to their priority by ordering the values in FOR in descending order. Subsequently, the highest-order relationships are selected, and the others are disregarded. The acquired relationships are computed and saved in the SOR cell. Algorithms 2 and 3 provide critical statistical features, including data attributes and distribution descriptions.
**Algorithm 2: FOR**1:  **for each pixel in** (*E* (*x,y*)) 2: **If**
*p* (*x*,y) = 0    {Black pixel at center}    **Then** Increase the frequency of occurrences at FOR (2,2) by 1 3: **If**
*p* (*x* + 1) = 0              {Black pixel at 0°}          **Then,**
Increase the frequency of occurrences at FOR (2,3) by 1 4:    **If**
*p* (*x* + 1, *y* − 1) = 0    {Black pixel at 45°}        **Then** Increase the frequency of occurrences at FOR (3,1) by 1 5:        **If**
*p* ((*x*, *y* − 1)) = 0                {Black pixel at 90°} **Then**
Increase the frequency of occurrences at FOR (2,1) by 1 6:        **If**
*p* ((*x* − 1, *y* − 1)) = 0            {Black pixel at 135°} **Then**
Increase the frequency of occurrences at FOR (1,1) by 1 7:     **If**
*p* (*x*, *y* − 1) = 0            {Black pixel at 180°} **Then** Increase the frequency of occurrences at FOR (2,3) by 1 8: **If**
*p* (*x* − 1, *y* + 1) = 0          {Black pixel at 225°}  **Then** Increase the frequency of occurrences at FOR (3,1) by 1 9:    **If**
*p* (*x*, *y* + 1) = 0            {Black pixel at 270°}  **Then** Increase the frequency of occurrences at FOR (2,1) by 1 10:    **If**
*p* (*x* + 1, *y* + 1) = 0            {Black pixel at 315°}  **Then**                         Increase the frequency of occurrences at FOR (1,1) by 1       **End**


**Algorithm 3: SOR**
1:   Sort *R*_1_ = FOR (*x*, *y*)↓ 2:   For each pixel in (*E* (*x*, *y*)) 3: If *E* (*x*, *y*) = Black                  **Then**                *R*_2_ = Relationships of neighborhood two pixels in *E* (*x*, *y*)) 4:      Compare (*R1*, *R2*) 5:                 Connected cell in SOR = SOR + 1, End

#### 3.2.2. Interest Point Features

This work uses the speeded up robust feature (SURF) to identify an image’s interesting points. SURF is a resilient representation approach invariant to translation, rotation, and scaling. This descriptor is used to find the similarity between different interesting points. The entry of an integral image ∫(x,y) at a location (*x*,*y*)*^T^* is used to represent the sum of all pixels in the input image I(x,y) within a rectangular region formed by the origin and (*x*,*y*)*^T^*. See Equation (11).

(11) $$\int \left(x,y\right)=\sum_{i=0}^{i\leq x}\sum_{j=0}^{j\leq y}I\left(i,j\right)$$

Additionally, the Hessian matrix is used to identify blob-like formations at regions where the determinant is optimal. The Hessian matrix *H* (*p*, *σ*) at point *p* = (*x,y*)*^T^* and scale *σ* = 3 is shown as Equation (12):

(12) Hp,σ=Lxxp,σLxyp,σLxyp,σLyyp,σ

where *L_xx_* (*p*, *σ*) is the convolution of the second-order derivative of the Gaussian $\frac{\partial g(x,\sigma )}{\partial x}$ with image ∫(x,y) at point p and is similar to $L_{xy}$ (*p*, *σ*) and *L_yy_* (*p*, *σ*).

#### 3.2.3. Curvelet Transformation (CT)

CT is a multiscale pyramid with several orientations and placements at each length and is needle-shaped at a small scale. CT was produced in recent years to address the inherent limits of conventional multiscale representations, which describe curve-like edges with a limited number of coefficients compared to wavelets far from optimal [46]. The CT technique captures the curved edge of characters in an Arabic script. The CT is mathematically described as Equations (13) and (14):

(13) $$\mathrm{CT}=\int_{R}^{0}f\left(x\right)\overset{-}{W_{a,b,\theta }}\left(x\right)dx$$

(14) $$W_{a,b,\theta }\left(x\right)=W_{a}\left(R_{\theta }\left(x-b\right)\right)$$

where $W_{a,b,\theta }\left(x\right)$ are the wavelet coefficients; a is the number of levels in the wavelet pyramid (a = 4); b = [3 4 4 5] represents location scalar parameters; *θ* is an orientation parameter $\theta \in \left[0,2\pi \right]$; Rθ=cosθsin⁡θsin⁡θcos⁡θ and is the rotation matrix with angle *θ*.

### 3.3. Feature Fusion

**Figure 8:**
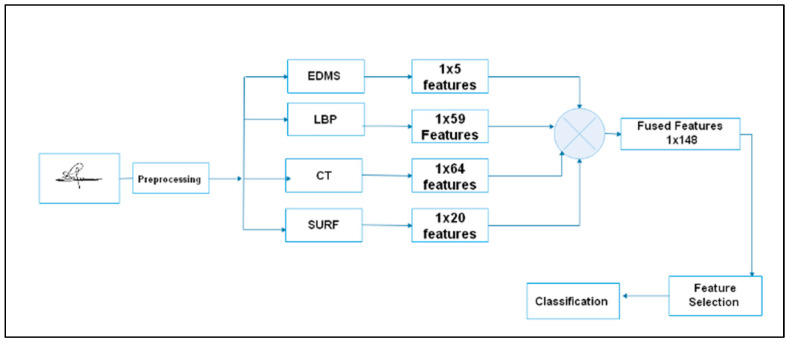
A strategy of feature fusion.

The precision of signature classification can be improved by extracting appropriate features. A method for fusing hybrid features is proposed to solve the restriction of a single feature extraction technique, as shown in Figure 8. Feature fusion combines several feature vectors to generate the final feature vector, which involves complementing each other’s advantages to obtain more robust and accurate outcomes [47]. The ESCF technique converts the feature matrix into a feature vector that describes the signature and can reduce error rates. ESCF is simple to implement, does not cause the loss of information, and has no impact on computational efficiency.

Let A and B be two feature spaces specified on pattern space Ω. For an arbitrary sample ξ ∈ Ω, the associated feature vectors are α ∈ A with n-dimensional features and β ∈ B with m-dimensional features; the Serial Fused feature of ξ is defined as γ=αβ with dimension (m+n). The mathematical description of the fusing process is based on Equation (15).

(15) $$F(v)=\left\lceil \begin{array}{c} {CT}_{1\times N}\\ {EDM}_{1\times N}\\ {SURF}_{1\times N}\\ {LBP}_{1xN} \end{array}\right\rceil$$

where F(v) is the final fused vector of 1 × sum(N) dimensions for all samples.

### 3.4. Feature Selection

Feature selection has been a productive area of research in intelligent algorithms and machine learning, which is undoubtedly essential. Feature selection eliminates attributes that may negatively affect the performance of classifiers, such as irrelevant, redundant, or less informative features. As indicated in the preceding section, a simple concatenated fusion technique combines various statistical features to generate an additional dimension that can identify skilled forgeries and genuine signatures with high accuracy.

The problem with combining characteristics without considering correlation and discrimination is that the resulting feature vector cannot detect a skilled forgery. Furthermore, fused features from multiple approaches may provide high-dimension features that could influence the verification process. As a result, a feature selection approach is necessary to minimize the number of features and remove data correlations.

GA has achieved success in many applications. GA can handle more complicated problems than neural networks and specializes in identifying an appropriate feature for a given class. However, automating the design of such fitness functions is still an open challenge. Adopting simple and effective fitness functions is a critical issue for GA.

**Figure 9:**
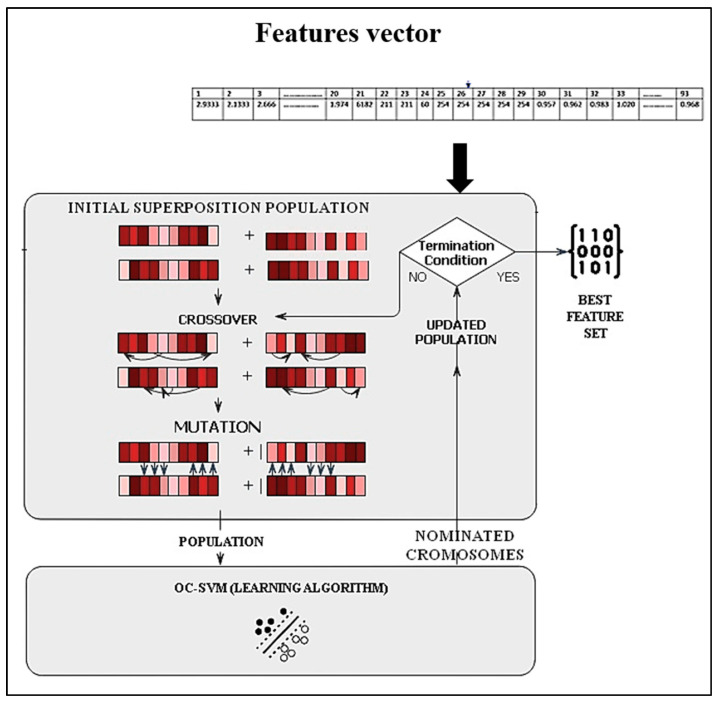
Flowchart of feature selection.

In this work, GA is used with one class support vector machine (OC-SVM) classifier to discover the genes with the highest predictive performance. Meanwhile, OC-SVM is employed for the classification. This proposal is one of the valuable contributions to reducing the issue of complexity and extending search spaces. Figure 9 shows the flowchart of the GA-OCSVM.

The procedure starts by randomly generating an initial population.

- The initial population size is created and set to 10.
- Calculate and assign a score of the fitness value to each member of the current population. These values are regarded as the raw fitness scores. The fitness function of each individual is determined by evaluating the OC-SVM using a training set. As a result, the fitness function containing classification precision is utilized in this study, as described in Equation (16).
  (16) $$fittness\left(f\right)=Max(accuracy\left(f\right))$$
  where accuracy (f) is the accuracy of the classifier for the subset selection of features expressed by f.
- Select members, known as parents, according to their expectations. Some individuals in the present population with maximum fitness levels are selected as elite (the subset with the best classification precision). These elite members are transmitted to the following population.
- Generates offspring from the selected parents. Offspring are produced by combining the vector entries of two parents (crossover). A uniform crossover with a crossover rate of 0.8 is employed.
- Low-frequency offspring introduce variety into a single-parent population (mutation). A uniform mutation method is selected with a mutation rate of 0.2.

The roulette wheel method is applied to randomly cross and mutate the Chromosome, which keeps the selective pressure in the center rather than at the extremes. In roulette wheel selection, the circular wheel is divided into n pies, where n equals the number of individuals. Based on their fitness level, each individual receives an appropriate circular piece. The wheel is rotated at a defined circumferential point. The region of the wheel just forward of the setpoint is referred to as the parent. The same method is followed for the next generations. The probability *Pi* of the individual is defined as Equation (17).

(17) $$Pi=\frac{f_{i}}{S}$$

where $S=\sum_{i=1}^{n}f_{i}$, *n* is the size of the population, and $f_{i}$ is the fitness of individual i.

- Individuals with higher fitness are more likely to be selected for reproduction.

The 110 features are selected as optimal features, a collection of discriminant characteristics. These distinguishing characteristics are fed into the classifier for verification.

### 3.5. One-Class Classification

**Figure 10:**
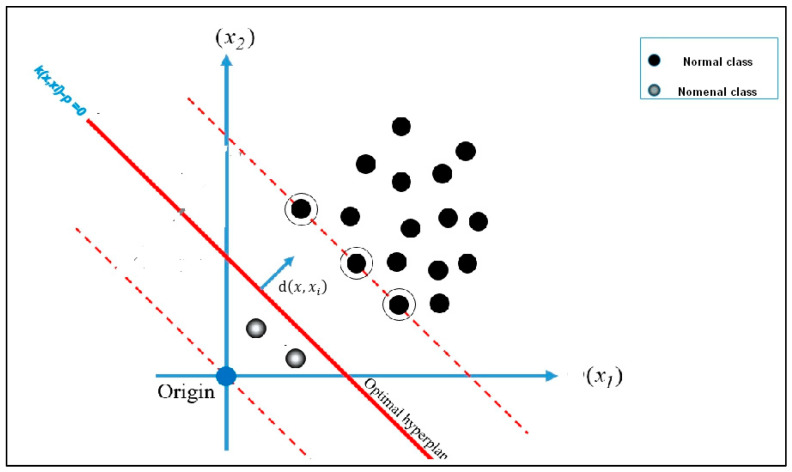
One-class support vector machine.

One-class classification (OCC) is used to solve the issue of an imbalanced database of signatures in the real world, where the authentic signature is only generated, and the forged signature is absent, which indicates that the original signer is incapable of forging the signature. This research employs OC-SVM to address these issues. The OC-SVM can successfully deal with the positive samples in the training set. The appropriate distance used by the radial basis function kernel (RBF kernel) must be specified to train the OC-SVM. OC-SVM is developed in two phases. First, one-class information (normal class) trains a classifier to distinguish genuine instances. The classifier rejects the samples belonging to unknown classes and classifies them as forgeries, as shown in Figure 10. A hypersphere with the shortest radius is constructed around the positive class data, which encloses approximately every point in the dataset. According to the parameter RBF γ, the hypersphere is defined by Equation (18).

(18) $$K\left(x_{i},x\right)=e^{-\gamma \left(d\left({x,x}_{i}\right)\right)}$$

where $d\left({x,x}_{i}\right)$ is the distance between the original images *x* and the target samples *x_i_* (or positive), and γ = 0.07 is the deviation parameter of the kernel function. The OC-SVM decision function is shown in Equation (19).

(19) $$f\left(x\right)=sign(\sum_{i=1}^{N}\alpha_{i}K\left(x_{i},x\right)-\rho ),$$

$$0\leq \alpha_{i}\leq \frac{1}{v}$$

where *N* is the number of training instances, ρ is the distance of the hypersphere from the origin, $\alpha_{i}$ denotes the Lagrange multiplier for each distance, and *v* = 0.01 represents the trade-off between maximizing the data points (encompassed by the hypersphere) and reducing the hypersphere’s distance from the origin. If the decision value of the sample is more significant than zero, we conclude that the target is a positive class; otherwise, it is a negative class.

## 4. Experimental Results and Analysis

The suggested model was evaluated on three databases: SID Arabic signatures, CEDAR, and UTSig [48,49,50]. The model was constructed in MATLAB^®^2021a on an Intel^®^ CoreTM i5-8300H CPU @2.30 GHz.

### 4.1. Experiments and Evaluation of Preprocessing

The proposed preprocessing strategies’ performance is appraised using image quality performance metrics such as mean square error (MSE) and peak signal-to-noise ratio (PSNR) in Equations (20) and (21). The assessment aims to illustrate the efficiency of image enhancement procedures on captured signature images.

The PSNR ratio is a quality measurement between the original image *I* and the enhanced image *j*. The high value of PSNR demonstrates the significant quality of the processed image. However, as mentioned above, more than this ratio is needed in the offline system because critical data may be lost during preprocessing. As a result, MSE is used to verify the image quality without losing key features. If the value of MSE is close to zero, the image quality is accepted. Conversely, the image loses its main attributes.

(20) $$\mathrm{PSNR}(I,J)=10\log_{10}R^{2}∕\mathrm{MSE}(I,J)$$

(21) $$\mathrm{MSE}(I,J)=\frac{1}{N*M}{\sum_{a=1}^{N}\sum_{b=1}^{m}(I(a,b)-J(a,b))}^{2}$$

M*N signifies the image size, and *I*(*a*, *b*) and *J*(*a*, *b*) denote the original and processed image pixel intensities, respectively. *R* is the maximum allowable pixel value. Six experiments were performed to show the impact and significance of each preprocessing method on the image, as shown in Table 2, Table 3 and Table 4.

Based on the MSE and PSNR results. The findings demonstrate that after applying the proposed preprocessing, the output images maintain the exact representation of the original images but with fewer pixels. It can be evidenced that the proposed preprocessing methods achieve an equilibrium between noise reduction and image data preservation.

### 4.2. Experiments and Evaluation of Verification

In this step, the performance of the proposed model is comprehensively evaluated. The verification is evaluated in two experiments: (a) features selection and extracted features without combining preprocessing; and (b) integration preprocessing with feature extraction and selection. FAR, FRR, and EER measurements were used to assess the model. FAR and FRR are two types of error measurements used to evaluate the performance of biometric systems. FRR is the percentage of authorized users whose request is incorrectly rejected. FAR is the percentage of unauthorized users who are mistakenly accepted, and EER specifies the point where the FRR and FAR are equal and stated as flowing equations.

(22) $$\mathrm{FAR}=\frac{no.\;of\;forged\;signatures\;classified\;as\;genuine}{Total\;no:\;forged\;signatures}\times 100$$

(23) $$\mathrm{FRR}=\frac{\begin{array}{c} no.\;ofgenuine\;signatures\;classified\\ as\;forged \end{array}}{Total\;no:\;genuin\;signatures}\times 100$$

(24) $$\mathrm{EER}=\frac{\mathrm{FRR}+\mathrm{FAR}}{2}$$

The proposed model was only trained on genuine samples with no forgeries to simulate the verification system in the real world, as shown in Table 5. The ratio validation of the model is 0.2. with random selected.

1. In the first experiment, the model performance was examined without preprocessing steps. This experiment aims to show the impact of preprocessing on verification accuracy. As shown in Table 6, the unsatisfactory verification results confirm that each preprocessing step significantly enhances image quality; this is what the second experiment proved.
2. The second experiment included all stages of the proposed model, including preprocessing, hybrid feature extraction, feature fusion, feature selection, and verification. The training phase was separately performed using three sets of genuine (G) samples. Table 7 displays the verification results on the SID Arabic database.

The suggested approach was also evaluated on CEDAR (English signature) and UTSIG (Farsi signature) to demonstrate its comprehensive performance. The model attained superb results on these databases, as shown in Table 8 and Table 9.

To compare the proposed algorithm results with existing signature verification techniques, Table 10, Table 11 and Table 12 show the results of the proposed model with state-of-the-art methods. From the results, it is clear that the performance of the presented model is good in terms of FAR and FRR using one-class training. The average error rate has been reduced to 10% in the SID database, 2–10% in the UTSIG database, and 3–7% in the CEDAR database.

### 4.3. Discussion

The proposed approach obtained superior FAR, FRR, and ERR on the three databases, particularly for skilled forgeries, which is the essential contribution of this study. Each stage of the model contributed to the increased precision. The preprocessing steps enhanced the verification results because all uninformative data and noise were removed. Moreover, the verification system’s supremacy is due to fused hybrid features and discrimination feature selection. The proposed feature extraction is advantageous because it combines multiple features to address the low intraclass difference between skilled forgery and genuine signatures, and the high intraclass difference between original signatures to the same writer. This combination maximizes the merit of each approach by complementing the advantages of other techniques, hence improving verification capabilities.

EDMs is a global textural descriptor used to analyze the entire image. Although EDMs were adequate for simple forgeries, they could not achieve high precision for skilled forgeries. For skilled forgers, LBP was more effective and accurate than EDMs. LBP is a local texture descriptor that describes a small part of the image and extracts more information. Some images may have local details that LBP could not figure out. In order to increase the detection percentage of skilled forgery, the SURF descriptor was used to add more distinct local features. SURF can detect and describe the interesting feature of the image. The key points in the picture include characteristics such as corners, edges, spots, and so on. The consistency of the key points can be helpful for performance. SURF outperforms SIFT in terms of performance and computational complexity.

**Figure 11:**
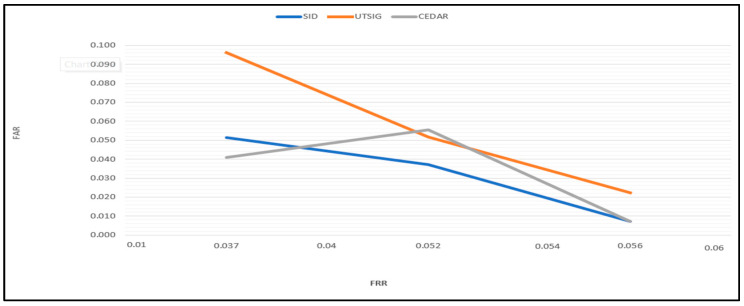
The accuracy in the plot curve.

Furthermore, the curvy lines in the Arabic characters were captured using a CT; CT accurately represents curved discontinuities. In addition, the feature selection strategy plays a crucial role in improving accuracy by removing insignificant characteristics. It also tackled the problem of correlation that may result from the feature fusion process, as illustrated in Figure 11.

Overall, the findings of the proposed methodology prove that the proposed method performs considerably better than recent signature verification approaches.

The complexity of the proposed model was assessed in this work using computing time. Each signature was processed in 0.01063 s, 0.01982 s, and 0.01544 s for the SID, CEDAR, and UTSIG databases.

## 5. Summary of the Scientific Work

The Arabic OSV system was presented using six stages: preprocessing, hybrid feature extraction and fusion, GA-OCSVM-based optimum feature selection, and OCC. This research suggests a multi-step process for preprocessing images, starting with image binarization, and moving on to denoising, segmenting, isolating, thinning the signature, and skeletonizing. Experiments yielded efficient results with high PSNR and low MSE. The suggested approaches significantly impacted verification processing time and accuracy.

Though the proposed method elicits and fuses four different statistical techniques, the ESCF fusion strategy has remained feasible regarding complexity. The best features were then selected using GA.

Additionally, OCC was employed to address the need for forgery signature samples in practical applications in the real world. The proposed model was implemented using the three databases mentioned earlier.

## 6. Conclusions

This paper proposed a signature verification model with four primary phases: preprocessing and hybrid feature extraction, followed by feature fusion. Finally, features selection and verification. The algorithm’s output was constructed with genuine and forged signature samples from three standard databases: SID-Signatures, CEDAR, and UTSIG. The suggested approaches significantly impacted the verification processing time and accuracy.

The proposed method combined four different statistical techniques. The best GA-based features were then identified for classification. Additionally, the proposed model employed OC-SVM to address the restriction of the current Arabic OVS regarding skilled forgery. The results revealed that the proposed system outperformed existing techniques. It improved the FAR by 10% on the SID-Arabic signature database without increasing the computation time. The experiment yielded 0.037 FRR, 0.039 FAR_skilled, 0.063 FAR simple, and 0.044 EER.

Moreover, the model was superior in enhancing the EER values of UTSig and CEDAR databases, which achieved 0.074 and 0.048, respectively. The FRR value could be enhanced by adding structural features in the future. Additionally, the accuracy of feature selection can be strengthened by improving crossover and mutation.

## Figures and Tables

**Table 1:** Summary of related work.

References	Features Used		Verification Approaches	Accuracy/EER
[29]	Global features and center of gravity features		Threshold technique	87% on the GPDS-960 database
[30]	Global features.		fuzzy-C means + threshold	9.2% EER on MCYT database
[31]	Global feature (entropy) and functional information features		SVM	97.81% n SVC2004 database
[23]	Deep CNN		SVM	12.83% EER on (GPDS) database 4.17% on (BRAZILIAN PUC-PR) database
[32]	Median of Medians (MoM) statistical dispersion measure (Δx)		Fuzzy similarity between test and training signature sample and threshold technique	0.11 ERR on (MCYT-100) database
				0.088 ERR on MCYT-330) database
				0.916 ERR on SVC database
				0.08 ERR on SUSIG database
[33]	Condensed Nearest Neighbors (CNN)		SVM	3.46% EER GPDS-960 dataset
[34]	global and grid features belonging		feature dimension and decision threshold	7.66% ERR on CEDAR database
				9.53 on MCYT database
[35]	Meta learning			4.70 ERR on GPDS dataset
				12.77 ERR on MCYT
				8.02 ERR on CEDAR
				6.7 ERR on Brazilian
[36]	Gray Level Co-occurrences Matrix (GLCM) and geometric features	SVM		2.33% MCYT,
				9.59% EER on GPDS synthetic
[37]	Structure- and direction-oriented features	Recurrent Neural Network (RNN)		GPDS-300 98.02%
				MCYT-75 99.39%
				BHSig260 Hindi 99.28%
				BHSig260 Bengali 99.37%
[38]	CNN method transfers learning SIFT + SVM			99.94% on
				SVM, 98.1 on 112 images were from IDRBT bank cheque dataset, used 50 images for testing
[39]	CNN			88% on signatures of 100 people, included 24 genuine signatures and 30 forged signatures

**Table 2:** Results of preprocessing on the SID database.

Binarization	Filtering	Segmentation	Isolation	Thinning	Skeletonization	PSNR	MSE
x	√	√	√	√	√	0.66473	55,796.55
√	x	√	√	√	√	48.22255	0.9791
√	√	x	√	√	√	59.33641	0.07576
√	√	√	x	√	√	48.22479	0.97859
√	√	√	√	x	√	48.21457	0.9809
√	√	√	√	√	x	48.21593	0.98059
√	√	√	√	√	√	64.85921	0.02124

**Table 3:** Results of preprocessing on the UTSIG database.

Binarization	Filtering	Segmentation	Isolation	Thinning	Skeletonization	PSNR	MSE
x	√	√	√	√	√	0.29984	60,687.15
√	x	√	√	√	√	48.29015	0.96397
√	√	x	√	√	√	58.16179	0.10013
√	√	√	x	√	√	48.29242	0.96347
√	√	√	√	x	√	48.27211	0.96799
√	√	√	√	√	x	48.27471	0.96741
√	√	√	√	√	√	62.94906	0.03297

**Table 4:** Results of preprocessing on the CEDAR database.

Binarization	Filtering	Segmentation	Isolation	Thinning	Skeletonization	PSNR	MSE
x	√	√	√	√	√	0.12439	63,189.01
√	x	√	√	√	√	48.14727	0.99622
√	√	x	√	√	√	60.87482	0.05316
√	√	√	x	√	√	48.16807	0.99146
√	√	√	√	x	√	48.16467	0.99223
√	√	√	√	√	x	48.1664	0.99184
√	√	√	√	√	√	72.40394	0.00374

**Table 5:** Division of database samples for modeling assessment.

Database	Phase	Genuine	Skilled Forgery	Simple Forgery
UTSIG	Training	Set1	0	0
		575 (5 × 115)		
		Set2		
		1150 (7 × 115)		
		Set3		
		1380 (10 × 100)		
	Testing	2530 (22 × 115)	690 (6 × 115)	4140 (20 × 115)
		2300 (20 × 115)		
		1955 (17 × 115)		
SID	Training	Set1	0	0
		700 (7 × 100)		
		Set2		
		1000 (10 × 100)		
		Set3		
		1200 (12 × 100)		
	Testing	3300 (33 × 100)	2000 (20 × 100)	2000 (20 × 100)
		3000 (30 × 100)		
		2800 (28 × 100)		
CEDAR	Training	Set1	0	
		275 (5 × 55)		
		Set2		
		385 (7 × 55)		
		Set3		
		550 (10 × 55)		
	Testing	1045 (19 × 55)	1320 (24 × 55)	
		935 (17 × 55)		
		770 (14 × 55)		

**Table 6:** Results of verification without preprocessing.

Database	FAR_Simple	FAR_Skilled	FRR	ERR
SID	0.077	0.065	0.190	0.130
UTSig	0.042	0.229	0.235	0.185
CEDAR	0.048		0.309	0.309

**Table 7:** Results of the proposed model on the SID database.

Training Signature Sample	FRR	FAR_Skilled	FAR_Simple	EER	STD *	Acc. (%)	Time (Sec.)
7G	0.032	0.061	0.072	0.049	0.017	95.099	86.8799
10G	0.026	0.041	0.070	0.041	0.014	95.929	82.2922
12G	0.037	0.039	0.063	0.044	0.007	95.583	85.0556

* STD represents the standard division.

**Table 8:** Results of the proposed model on the CEDAR database.

Training Signature Sample	FRR	FAR	ERR	STD	Acc. (%)	Time (Sec.)
5G	0.065	0.058	0.061	0.002	93.859	46.647
7G	0.067	0.034	0.051	0.008	94.926	48.5451
10G	0.056	0.041	0.048	0.004	95.179	52.3336

**Table 9:** Results of the proposed model on the UTSIG database.

Training Signature Sample	FRR	FAR_Skilled	FAR_Simple	ERR	STD	Acc. (%)	Time (Sec.)
5G	0.045	0.186	0.028	0.076	0.03	92%	97.6121
7G	0.055	0.178	0.024	0.078	0.02	92%	100.738
10G	0.052	0.162	0.030	0.074	0.02	93%	127.86

**Table 10:** Comparison of the results of the proposed approach and the state-of-the-art methods using the SID database.

References	Feature Type	Classifier	FAR- Simple	FAR- Skilled	FRR	EER
[48]	Geometric features + wavelet Transformation	(MLP)	0.0379	0.0895	0.1495	0.0984
[51]	Graphometric + geometric	NN	0.0745	0.1870	-	-
		HMM	0.295	0.3705		
		SVM	0.1385	0.1875		
[52]	Global feature	PMCM-BP/SVM	0.0835	0.0910	-	-
Proposed			0.063	0.039	0.037	0.044

**Table 11:** Comparison of the results of the proposed approach and the state-of-the-art methods using the UTSIG database.

References	Features Type	Classifier	FAR-Skilled	FRR	EER
[53]	ResNet CNN pretrained on Handwriting classification tasks	SVM	-	-	0.0980
[49]	Geometric features	SVM	0.1841	0.4170	0.2933
	(fixed-point arithmetic)				
[54]	DWT + Gabor filter	CNN	0.1365	0.3267	0.223
[55]	HOG+	Discriminative Deep Metric Learning (DDML)	0.1615	0.1896	0.1745
	DRT				
[56]	Gaussian Weighting Based Tangent	SVM	0.2495	0.0741	0.1618
	Angle (GWBTA) + Cylindrical Shape Context				
[57]	Statistical + shape based + Similarity based+ Frequency based	Binary Red Deer Algorithm (BRDA) feature selection + Naïve Bayes classifier	-	-	0.100
Proposed			0.162	0.052	0.074

**Table 12:** Comparison of the results of the proposed approach and the state-of-the-art methods using the CEDAR database.

References	Features Type	Classifier	FAR	FRR	EER
[58]	Histogram of oriented gradients (HOG)	SVM	-		0.2092
	LPB				0.0890
	LDF		0.0654	0.0581	0.0618
[59]	Chain code histogram	SVM	0.0784	0.0939	0.086
[60]	DWT + local quantized patterns (LQP)	SVM	0.0746	0.0786	0.0766
[61]	Local + Global	SVM	0.0743	0.0446	0.0595
[34]	Geometric feature	Threshold	0.0654	-	0.0766
[62]	DWT + multi-resolution box-counting (MRBC)	Gaussian process (GP)	0.0757	0.0643	0.07
[5]	pretrained DCNN(GoogLeNet) + NCA features selection	SVM	-	-	0.200
[63]	SNN	Threshold	0.0734	0.0694	0.0714
[64]	Interval type-2 fuzzy set (IT2FS	ELM (extreme learning machine) + SRC (sparse representation classifier	0.1054	0.1236	0.111
[65]	AlexNet	Decision Tree (DT)	-	-	0.079
Proposed			0.041	0.056	0.048

## Data Availability

Not applicable.
